# New insights into a microvascular invasion prediction model in hepatocellular carcinoma: A retrospective study from the SEER database and China

**DOI:** 10.3389/fsurg.2022.1046713

**Published:** 2023-01-06

**Authors:** Xingchang Wang, Yiling Fu, Chengzhan Zhu, Xiao Hu, Hao Zou, Chuandong Sun

**Affiliations:** ^1^ Department of Hepatobiliary and Pancreatic Surgery, The Affiliated Hospital of Qingdao University, Qingdao, China; ^2^ Department of Rehabilitation Medicine, Qilu Hospital of Shandong University (Qingdao), Qingdao, China

**Keywords:** hepatocellular carcinoma, microvascular invasion, predicting model, nomogram, SEER, external validation

## Abstract

**Background and Aims:**

The prognosis of liver cancer is strongly influenced by microvascular infiltration (MVI). Accurate preoperative MVI prediction can aid clinicians in the selection of suitable treatment options. In this study, we constructed a novel, reliable, and adaptable nomogram for predicting MVI.

**Methods:**

Using the Surveillance, Epidemiology, and End Results (SEER) database, we extracted the clinical data of 1,063 patients diagnosed with hepatocellular carcinoma (HCC) and divided it into either a training (*n* = 739) or an internal validation cohort (*n* = 326). Based on multivariate analysis, the training cohort data were analyzed and a nomogram was generated for MVI prediction. This was further verified using an internal validation cohort and an external validation cohort involving 293 Chinese patients. Furthermore, to evaluate the efficacy, accuracy, and clinical use of the nomogram, we used concordance index (C-index), calibration curve, and decision curve analysis (DCA) techniques.

**Results:**

In accordance with the multivariate analysis, tumor size, tumor number, alpha-fetoprotein (AFP), and histological grade were independently associated with MVI. The established model exhibited satisfactory performance in predicting MVI. The C-indices were 0.719, 0.704, and 0.718 in the training, internal validation, and external validation cohorts, respectively. The calibration curves showed an excellent consistency between the predictions and actual observations. Finally, DCA demonstrated that the newly developed nomogram had favorable clinical utility.

**Conclusions:**

We established and verified a novel preoperative MVI prediction model in HCC patients. This model can be a beneficial tool for clinicians in selecting an optimal treatment plan for HCC patients.

## Introduction

Primary liver cancer is one of the most prevalent cancers and is a major contributor to the global cancer mortality rate. Its morbidity and mortality rates are among the highest worldwide (1). Mortality from primary liver cancer is the second highest among malignant tumors and the fourth most common type of cancer in China (2). Annually, the number of new cases and deaths of primary liver cancer is about 466,100 and 422,000, respectively (3). Hepatocellular carcinoma (HCC) is the most prevalent pathological type of primary liver cancer and respects about 80% of all primary liver cancer worldwide (4).. To date, the most effective interventions for HCC are hepatectomy and liver transplantation. However, patient prognosis following these treatments remains relatively poor (5). Moreover, it has a recurrence rate of 70% 5 years after surgery (6, 7). Thus, despite the rapid development of HCC diagnosis and treatment, its high recurrence rate remains a considerable challenge (8).

Vascular invasion is strongly linked with tumor malignancy, disease recurrence, and poor patient prognosis. In individuals with HCC, this invasion may be divided into two subtypes: macrovascular and microvascular. Macrovascular invasion is typically identified by imaging, and patients experiencing this form of invasion often do not have the chance to undergo radical resection or liver transplantation. Alternatively, microvascular infiltrations (MVI) status is only available by pathological examination of surgical specimens. It is a nest of cancer cells in endothelium-lined vessels, which may be observed under a microscope and are usually present at the small branches of the portal vein in the surrounding liver tissues (9). MVI can result in the dissemination and metastasis of tumor cells within the liver, or even lead to metastasis to other parts of the body (10). Therefore, MVI is thought to be one of the main risk factor influencing tumor recurrence and survival (11), and it is often employed as a prognostic indicator to guide the choice of an appropriate treatment regimen in patients with both primary and recurrent HCC (12, 13). Meanwhile, emerging evidence has revealed that MVI assists in clinical decision-making. For instance, it was suggested that the precise preoperative prediction of MVI status can assist in identifying surgical resection margins to enhance patient outcomes (14, 15). Furthermore, individuals with MVI-positive HCC whose surgeries were accompanied by adjuvant intervention or targeted treatment had better overall survival (OS) than those who underwent surgery alone (16, 17). Hence, accurate prediction of MVI status is crucial for providing an efficient and successful intervention for HCC patients.

Unfortunately, MVI diagnosis is established only by histopathological assessment of surgical specimens following HCC resection or liver transplantation. At present, there is no effective or precise prediction method prior to surgery, which greatly limits the effect of preoperative assessment on surgical planning and patient outcome. Hence, finding a method to accurately and efficiently predict MVI is an urgent problem to be solved at present (18). A nomogram is a feasible and efficient tool that integrates and quantifies marked risk factors to predict patient outcome (19). Numerous studies have generated and verified different nomograms for MVI predictions. However, most of these nomograms were based on single-center studies that lack external verification, which puts into question the reliability and applicability of these models (20, 21). In addition, there are limited studies available on the risk assessment of MVI.

Hence, the purpose of the present study was to generate a novel, reliable, and adaptable nomogram to predict the incidence of MVI in HCC patients. This can aid clinicians in selecting suitable therapeutic measures for patients with MVI-positive HCC.

## Methods

### Data sources and patient population

Patient records were retrospectively obtained from the Surveillance, Epidemiology, and End Results (SEER) database, maintained by the US National Cancer Institute. Co-created by 18 registries throughout the United States, this database includes information on the prevalence of illness and outcomes for patients with tumors across approximately 28% of the country. Patient data included the following demographics: age, sex, ethnicity, country of birth, and specifics of the patient's tumor (including its histology and grade) and treatment (including details about any surgeries, radiation, or other interventions). Recently added data include AJCC stage, surgical parameters, tumor size, and lymph node involvement. In addition to a large patient pool and improved data accuracy, this database contains tumor profiling specimens.

**Figure 1:**
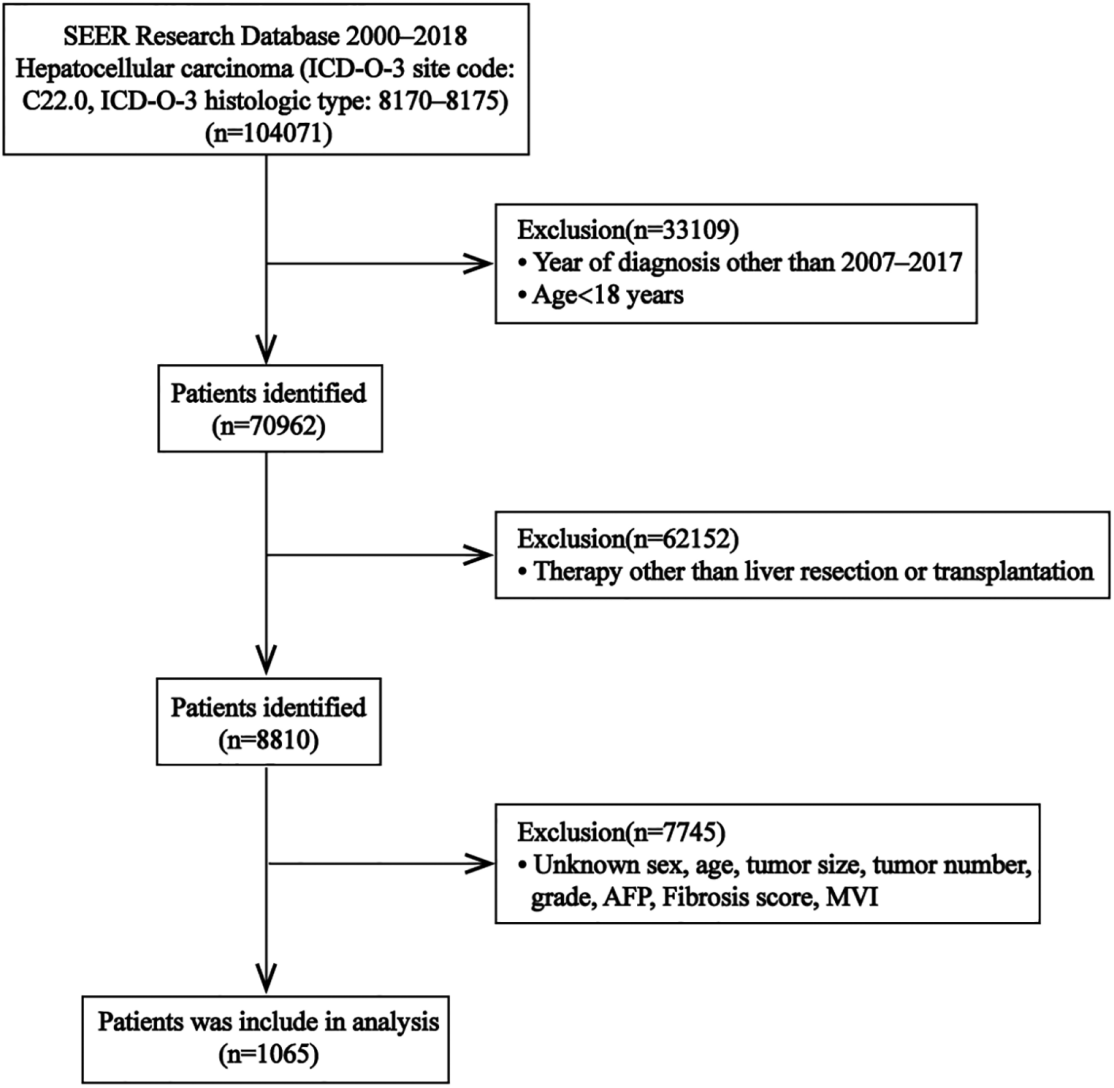
Flow chart for selection of the study population from SEER database. Abbreviations: SEER, Surveillance, Epidemiology, and End Results; ICD-O-3, International Classification of Diseases for Oncology, 3rd edition; MVI, microvascular invasion; AFP, alpha fetal protein.

Using the SEER stat program (SEER*Stat 8.4.0.1), we were able to access the SEER database and obtain records of HCC patients diagnosed between 2007 and 2017. The inclusion criteria were shown below: (I) patients with HCC (ICD-0–3:8170–8175), and the primary tumor site was liver; (II) age at diagnosis ≥18 years; (III) those who undergo liver resection or liver transplantation with postoperative histopathological confirmation. The following patients were excluded from the analysis: (I) pathologically confirmed other than HCC; (II) those with MVI status not determined *via* histopathological evaluation; (III) patients with HCC with macrovascular or extrahepatic infiltration; and (IV) those with missing clinical information. The selection process is illustrated in Figure 1. Ultimately, we acquired information on 1,063 patients. Subsequently, we randomly spilt all cases into either a training cohort and an internal validation cohort in a 7:3 ratio.

Next, we assessed the general applicability of the proposed model. We employed an external validation cohort composed of 293 Chinese patients with HCC who received treatment at the Affiliated Hospital of Qingdao University from January 2017 to December 2019 using the same patient selection criteria as mentioned above. Our study was reviewed and approved by the Ethics Committee of the Affiliated Hospital of Qingdao University (approval no: QYFY WZLL 27357) and was performed according to the latest version of the Declaration of Helsinki. Written informed consent was waived due to the retrospective nature of the study.

### Clinical variables and pathological characteristics

Clinicopathological features, such as patient age, sex, tumor size, tumor number, MVI status, histological grade, alpha-fetoprotein (AFP), and fibrosis score, were acquired from the SEER database and the Affiliated Hospital of Qingdao University. Fibrotic stage was divided into F0–4 (no fibrosis to moderate fibrosis) and F5–6 (severe fibrosis) based on the Ishak score from the SEER database.

### Statistical analysis

Categorical data, analyzed using the chi-square or Fisher's exact tests, are presented statistically as numbers of cases with percentages. The Wilcoxon rank sum test was used to analyze continuous data, which were provided as means with interquartile ranges (IQRs). To assess the MVI risk factors, we conducted univariate analyses on the training cohort information. All data (*P* < 0.05) in the univariate analyses were subjected to multivariate analysis to identify independent MVI risk factors, and an MVI prediction nomogram was generated according to the multivariate analysis results.

We calculated the concordance index (C-index) using 1,000 bootstrap samples for measurement discrimination to evaluate the predictive performance of the nomogram. Using calibration plots, we checked how well our predictions matched the real-world data. Using decision curve analysis (DCA), we were able to assess the nomogram's clinical efficacy by quantifying their net benefit at different cutoff probabilities. Statistical significance was set at *P* < 0.05. All analyses were performed using R v3.6.3.

## Results

### Clinicopathological profiles

The SEER database was mined for 1,065 patients with HCC. To develop and verify the nomogram, the patients' data were randomized into a training cohort (*n* = 739) and an internal validation cohort (*n* = 326). The external validation cohort consisted of 293 Chinese patients with HCC. The training cohort included 536 males and 203 females, the internal validation cohort consisted of 243 males and 83 females, and the external validation cohort consisted of 214 males and 79 females. The median tumor sizes were 3.6 cm (range 2.4–5.5), 3.5 cm (range 2.3–5.5), and 3.5 cm (range 2.5–5.5) in the training, internal validation, and external validation cohort, respectively. Histopathological MVI detection was positive in 141 of 739 patients (19.1%) in the training cohort; 68 of 326 patients (20.9%) in the internal validation cohort; and 158 of 293 patients (53.9%) in the external validation cohort. Patient demographics and clinicopathological profiles of the three patient populations are summarized in Table 1.

**Table 1:** Baseline demographics and clinical characteristics of the training, internal validation, and external validation cohort.

Baseline Characteristics	Number (%)/Median (IQR)*			*P* value
	Training Cohort (*n* = 739)	Internal Validation Cohort (*n* = 326)	External Validation Cohort (*n* = 293)	
Sex				0.792
Male	243 (74.5)	536 (72.5)	214 (73.0)	
Female	83 (25.5)	203 (27.5)	79 (27.0)	
Age(years)				0.802
<60	131 (40.2)	289 (39.1)	121 (41.3)	
≥60	195 (59.8)	450 (60.9)	172 (58.7)	
Tumor size (cm)	3.50 (2.30, 5.20)	3.60 (2.40, 5.50)	3.50 (2.50, 5.50)	0.655
Tumor number				<0.001
Multiple	71 (21.8)	155 (21.0)	30 (10.2)	
Single	255 (78.2)	584 (79.0)	263 (89.8)	
MVI				<0.001
Negative	258 (79.1)	598 (80.9)	135 (46.1)	
Positive	68 (20.9)	141 (19.1)	158 (53.9)	
Grade				<0.001
I	90 (27.6)	170 (23.0)	10 (3.4)	
II	180 (55.2)	444 (60.1)	148 (50.5)	
III	54 (16.6)	116 (15.7)	125 (42.7)	
IV	2 (0.6)	9 (1.2)	10 (3.4)	
AFP				0.001
Negative	137 (42.0)	286 (38.7)	150 (51.2)	
Positive	189 (58.0)	453 (61.3)	143 (48.8)	
Fibrosis score				0.452
F0–4	131 (40.2)	298 (40.3)	130 (44.4)	
F5–6	195 (59.8)	441 (59.7)	163 (55.6)	

Abbreviations: MVI, microvascular invasion; AFP, a-fetoprotein.

* Median with interquartile range are shown for quantitative variables, whereas counts with proportions are shown for categorical variables.

### Independent risk factors associated with MVI Status

Tumor size, tumor number, histological grade, and AFP were all shown to have significant correlations with MVI based on the univariate analyses of clinicopathological characteristics between the MVI-positive and negative patient groups (Table 2). Furthermore, using the multivariate analysis, all the aforementioned variables were shown to be independent risk factors of MVI (Table 3).

**Table 2:** Univariate ordinal logistic analysis for MVI status in the training cohort (*N* = 739).

Factor	Number (%)/Median (IQR)*		*P* value
	Negative (*n* = 598)	Positive (*n* = 141)	
Sex			0.79
Male	435 (72.74%)	101 (71.63%)	
Female	163 (27.26%)	40 (28.37%)	
Age (years)			0.869
<60	233 (38.96%)	56 (39.72%)	
≥60	365 (61.04%)	85 (60.28%)	
Tumor size (cm)	3.3 (2.3, 5.0)	4.8 (3.2, 7.0)	<0.001
Tumor number			0.015
Multiple	136 (22.74%)	19 (13.48%)	
Single	462 (77.26%)	122 (86.52%)	
Grade			<0.001
I	159 (26.59%)	11 (7.8%)	
II	355 (59.36%)	89 (63.12%)	
III	77 (12.88%)	39 (27.66%)	
IV	7 (1.17%)	2 (1.42%)	
AFP			<0.001
Negative	255 (42.64%)	31 (21.99%)	
Positive	343 (57.36%)	110 (78.01%)	
Fibrosis score			0.081
F0–4	232 (38.8%)	66 (46.81%)	
F5–6	366 (61.2%)	75 (53.19%)	

*P* value: categorical variables-*χ*^2^test or Fisher's exact test; continuous variables-Wilcoxon rank sum test.

Abbreviations: HCC, hepatocellular carcinoma; MVI, microvascular invasion; AFP, a-fetoprotein.

* Median with interquartile range are shown for quantitative variables, whereas counts with proportions are shown for categorical variables.

**Table 3:** Multivariable analysis of predictors associated with MVI in the training cohort (*N* = 739).

Factor	OR	95% CI	*P* value
Tumor size (cm)	1.005	1–1.009	0.04
Tumor number			
Multiple			1 (reference)
Single	1.101	1.029–1.178	0.005
**Grade**			
I			1 (reference)
II	1.123	1.049–1.202	0.001
III	1.262	1.152–1.383	<0.001
IV	1.075	0.832–1.39	0.58
**AFP**			
Negative			1 (reference)
Positive	1.117	1.054–1.183	<0.001

Abbreviations: AFP, a-fetoprotein; OR, (odds ratio) = e^Estimate^; CI, confidence interval.

### Construction and verification of an MVI prediction nomogram

**Figure 2:**
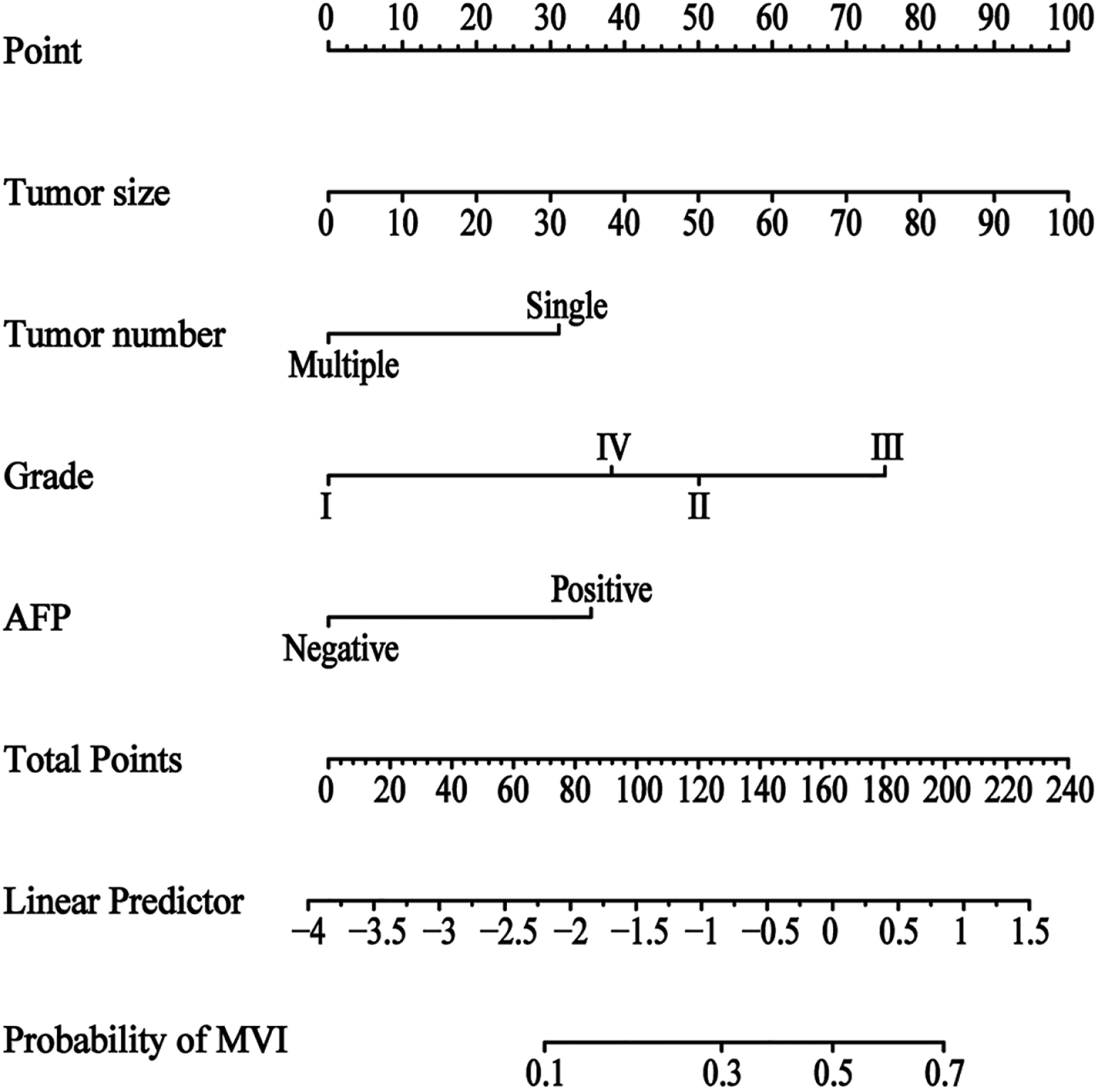
Nomogram for predicting the status of microvascular invasion preoperatively in patients with hepatocellular carcinoma. The MVI nomogram was built by incorporating tumor size, tumor number, histological grade and AFP. Locate the patient's characteristic on a variable row and draw a vertical line straight up to the points’ row (top) to assign a point value for the variable. Adding up the total number of points and drop a vertical line from the total points’ row to obtain the probability of predictive outcomes. Abbreviations: MVI, microvascular invasion; AFP, a-fetoprotein.

**Figure 3:**
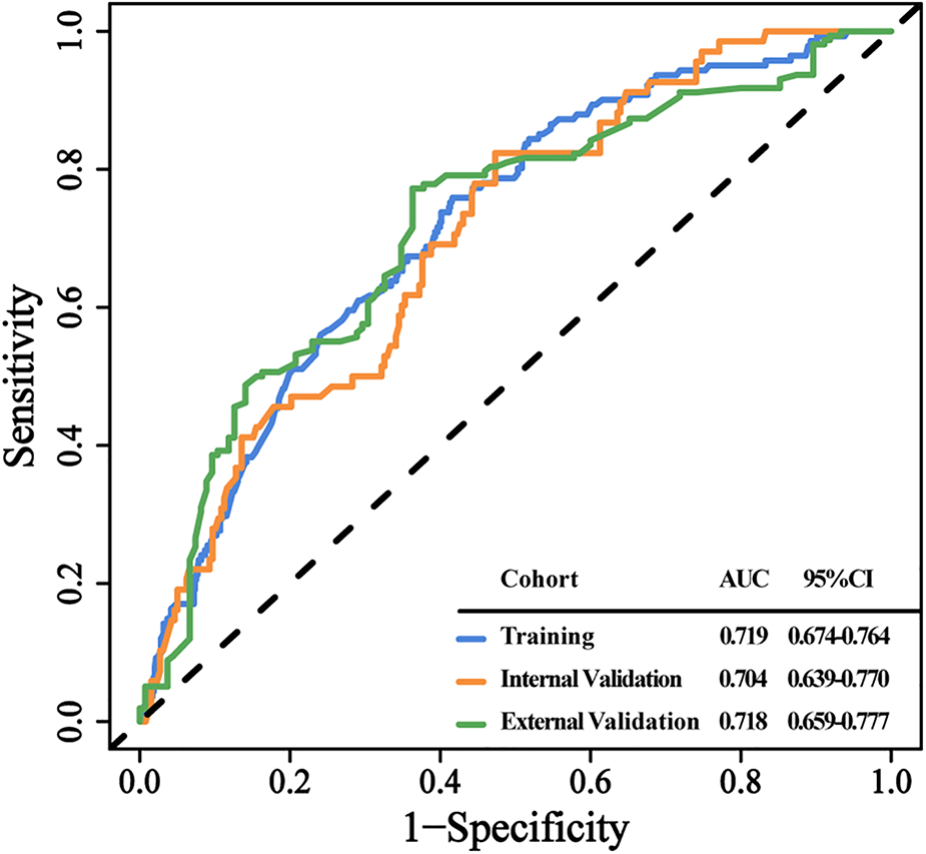
The discrimination of the clinical prediction model in the 3 data cohorts. ROC curves for MVI probability in the training (**A**), internal validation (**B**), and external validation cohort (**C**), respectively. Abbreviations: ROC, receiver operating characteristic curve. AUC, area under curve.

Using prognostic indicators, including tumor size, tumor number, histological grade, and AFP, we built a nomogram to predict MVI (Figure 2). We can predict the incidence of MVI by employing this nomogram to compute the total points for individual patients. In the training cohort, the C-index was 0.719 (95% CI: 0.674–0.764), in the internal validation cohort, it was 0.704 (95% CI: 0.639–0.77), and in the external validation cohort, it was 0.718 (95% CI: 0.659–0.777) Figure 3). Based on these data, nomogram exhibited excellent performance in delineating between negative and positive MVI incidence.

**Figure 4:**
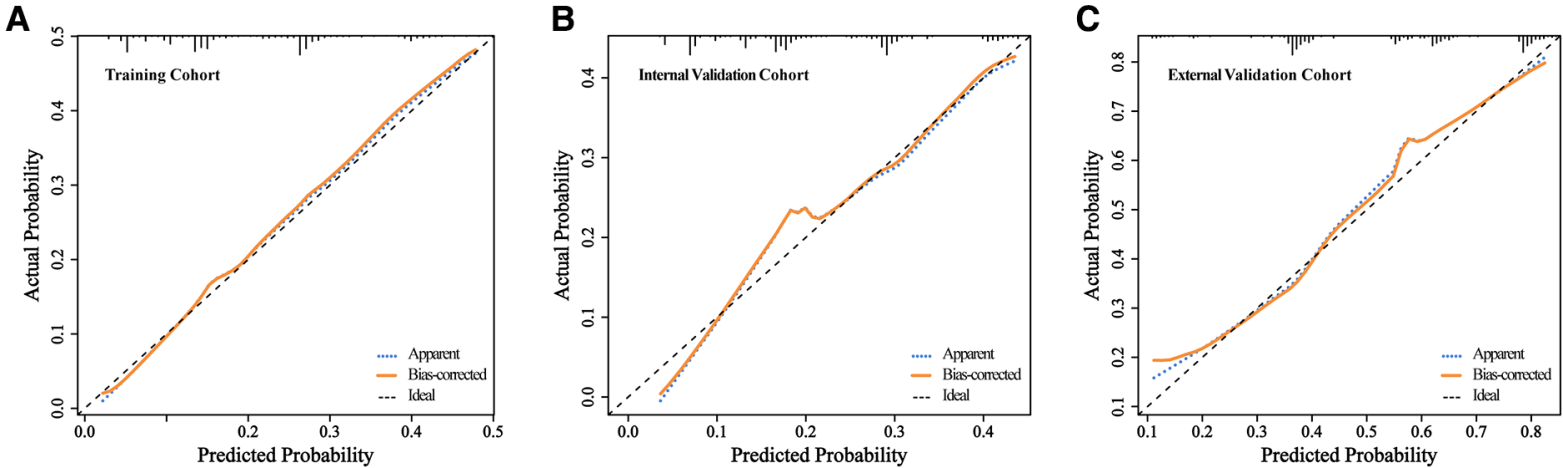
The calibration of the clinical prediction model in the 3 data cohorts. Calibration curves for predicting patient MVI at each time point in the training (**A**), internal validation (**B**), and external validation cohort (**C**), respectively. Model-predicted MVI is plotted on the x-axis, and actual MVI is plotted on the y-axis. The calibration curves of the nomogram based on internal validation with a bootstrap resampling frequency of 1,000. A plot along the 45- degree line (dotted black line) would indicate a perfect calibration model in which the predicted probabilities are identical to the actual outcomes.

**Figure 5:**
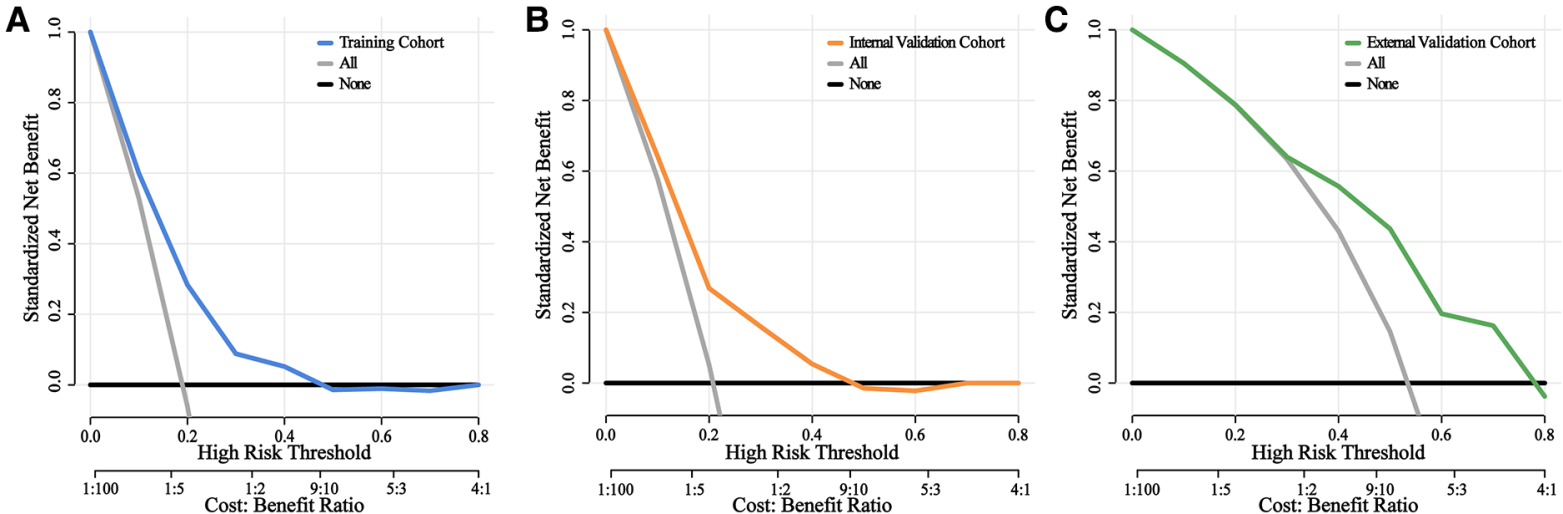
Decision curves of the nomograms for predicting status of MVI. DCA curves of the nomogram at each time point in the training (**A**), internal validation (**B**), and external validation cohort (**C**), respectively. The horizontal solid black line represents the hypothesis that no patients experienced the status of MVI, and the solid gray line represents the hypothesis that all patients met the endpoint. The blue, orange, and green line, represents the net benefit of the nomogram at different threshold probabilities. The net clinical benefit was calculated as the true-positive rate minus the weighted false-positive rate.

Furthermore, we constructed calibration plots to assess the calibration of our prediction model. Based on our analysis, there was excellent agreement between the actual and predicted likelihood of MVI among HCC patients in the training, internal validation, and external validation cohorts, respectively (Figures 4A–C). DCA was employed for the evaluation of total benefit under varying clinical decisions at different threshold likelihood. According to the DCA results of training and internal validation cohort (Figures 5A,B), it is useful to employ this nomogram to predict MVI between threshold probabilities of 0 and 0.4; and the DCA results of external validation cohort (Figure 5C) revealed that it is useful to employ this nomogram to predict MVI between threshold probabilities of 0 and 0.8, thus, indicating a satisfactory clinical application of the developed nomogram. To better understand their significance, nomogram clinical impact curves for MVI prediction were plotted (Supplementary Figures S1A–C). Based on our results, the model exhibited a substantial predictive value.

## Discussion

The major factor behind the poor outcome of HCC patients is the high relapse rate of this disease (9, 22). As one of the major contributor to HCC recurrence, MVI can strongly influence tumor cell intrahepatic metastasis *via* the portal circulation (23) and induce tumor recurrence following operation (24). Hence, MVI is typically considered an essential prognostic indicator for HCC following surgery. Additionally, MVI also impacts preoperative decision-making. In 2017, Zhao et al. demonstrated that anatomical hepatectomy enhances recurrence-free survival in MVI-positive patients (25). Mazzaferro et al. revealed no obvious variability in the 5-year OS rate following liver transplantation between the Milan and MVI-negative Up-to-seven criteria usage (26). However, MVI can only be detected *via* histopathological evaluation following surgical resection (27), which limits its clinical application. Therefore, it is crucial to preoperatively predict MVI for guiding clinical decision-making and improving patient prognosis.

Using a retrospective investigation of the SEER and Affiliated Hospital of Qingdao University databases, we established and verified a novel preoperative prediction model for MVI in HCC patients. The nomogram accurately identified patients with negative and positive preoperative MVI. Furthermore, the estimated likelihood was comparable with the true incidence of MVI. Herein, we demonstrated that tumor size, tumor number, histological grade, and AFP were markedly related to MVI occurrence. In the multivariate analysis, the amplitude of the odds ratios statistically significant. But no value had an independent predictive contribution over 26%, they had worthwhile predictive power together. The relationship between some of these factors and the MVI has also been verified in other studies. A prior report suggested that MVI incidence increased with tumor size in HCC patients (≤3 cm, 25%; 3.1–5 cm, 40%; 5.1–6.5 cm, 55%; >6.5 cm, 63%) (28). Kim et al. (29) and Siegel et al. (30) revealed that tumor sizes over 2 or 3 cm are risk indicators of MVI, respectively. Our investigation corroborates the aforementioned reports. Multiple studies have suggested that an elevated AFP level is independently associated with MVI incidence (31, 32), which is in accordance with our results. Histological grade represents the HCC differentiation status. Preoperative HCC diagnosis usually requires liver biopsy. Once HCC is confirmed, further information on HCC differentiation can be obtained at the same time. Yao et al. revealed that tumor size and histological grade are independent factors related to MVI (33), which is in accordance with our data. Notably, to date, there is no consensus on the relationship between tumor quantity and MVI. Wang et al. reported that multiple tumors strongly indicate MVI (10). Alternatively, Yan et al. revealed that solitary nodules were distinctly related to MVI (34). Based on our multivariate analysis, patients with HCC with a single tumor were more vulnerable to MVI formation. However, this finding requires further validation.

Numerous investigations have explored the risk factors for predicting MVI. For instance, Mao et al. reported that preoperative large tumor diameter, AFP over 20 ng/ml, and total bilirubin level > 23 umol/L were strongly related to MVI. Moreover, they constructed an MVI prediction nomogram using these factors (21). Deng et al. established a nomogram that combined tumor size, preoperative AFP level, and neutrophil-to-lymphocyte ratio to predict MVI (35). Lin et al. generated a nomogram that included the intratumoral artery, tumor type, tumor diameter, and AFP level, and it exhibited satisfactory performance in predicting MVI occurrence in patients with HCC (36). However, the majority of previous studies were single-center investigations and employed relatively small sample populations. It is possible that such study characteristics may have impeded the reliability, limited generalizability, and external applicability of their models. Relative to those studies, our MVI prediction model was established and validated using a multicenter platform, which included a relatively large sample population, and it demonstrated strong predictability among different populations (American and hospitalized Chinese populations). Hence, our model is more reliable and applicable.

Currently, there is much interest in MVI prediction models based on radiomics. To predict MVI, Xu et al. developed a novel computational method that integrates extensive clinico-radiologic and radiomic data, including AST, AFP, tumor margin, growth pattern, capsule, peritumoral enhancement, radio-genomic venous invasion, and radiomic score, with promising results (37). To predict MVI status in HCC, Hyun et al. constructed a nomogram that includes the tumor-to-normal liver standardized uptake value ratio on FDG PET/CT, clinical tumor size, and AFP (38). Zhang et al. used some Gd-EOB-DTPA MRI features and biochemical indicators to develop a new diagnostic scoring system to predict MVI, consisting of maximum tumor diameter, peritumoral hepatobiliary phase reduced intensity, incomplete capsule, apparent diffusion coefficient, and [alkaline phosphatase (U/L) + gamma-glutamyl transpeptidase (U/L)]/lymphocyte count (×109/L) ratio (39). However, the accuracy and practicality of these models are doubtful because of the lack of uniformity in radiomics and overreliance on the judgment of diagnostic radiologists (40). Additionally, certain specialized radiological parameters are often not understood or used by clinicians. In contrast, we built our model using data from regular laboratory tests, which are easily accessible, standardized, and manageable. It is also simple and straightforward to compare and contrast the data from different sources. Thus, it is clear that our model is not only better in terms of standardization and popularization but also facilitates clinical use.

Although our study has many advantages, it also has certain limitations. First, owing to its retrospective nature, this study may suffer from potential bias. Hence, further studies using prospective patient data are required. Second, owing to the limited clinical information acquired from the SEER database, many reported clinical risk factors known to be associated with MVI, such as platelet count (32), neutrophils (10) and total bilirubin level (21), were not included in this research. This may have negative effect on the prediction ability of the model, to a certain extent. Third, at present, radiographic diagnosis with LIRADs criteria in appropriate risk groups gradually replaced biopsy as the standard diagnostic method for HCC. However, the SEER database lacks relevant imaging information, so it is necessary to collect relevant imaging information in the following research to further improve the nomogram. Fourth, this model was established and verified using the SEER database, and external validation was done in a single-center in China. However, the applicability of this model to individuals of other ethnicities or racial is uncertain. Thus, external verification using other centers is required prior to the widespread use of this model in clinics.

In summary, we established and verified a novel, reliable, and adaptable preoperative prediction model for MVI in HCC patients. Our model was composed of four routine laboratory parameters (tumor size, tumor number, histological grade, and AFP), and it demonstrated superior delineation properties in terms of MVI diagnosis. This model can potentially aid clinicians in establishing the individualized risk of patients with MVI development, which in turn, can assist in proper treatment application.

## Data Availability

The datasets presented in this study can be found in online repositories. The names of the repository/repositories and accession number(s) can be found in the article/**Supplementary Material**.
