# Supplementary material for: New insights into a microvascular invasion prediction model in hepatocellular carcinoma: A retrospective study from the SEER database and China
Source: Front Surg. 2023 Jan 6;9:1046713. doi: 10.3389/fsurg.2022.1046713 (PMC9853393; doi:10.3389/fsurg.2022.1046713)
Supplement: Supplementary file 1 [file Datasheet1.docx]

Supplementary Material

# Supplementary Figures

**
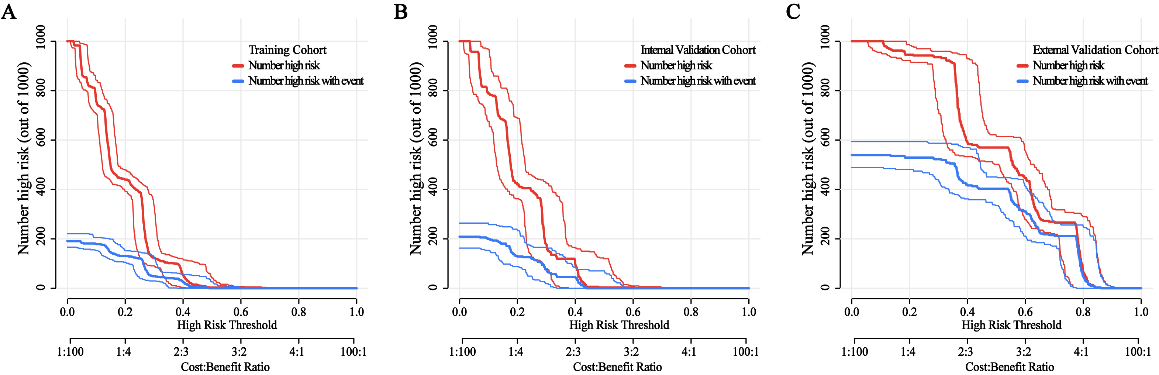
**

**Supplementary** **Figure 1.** Clinical impact curves of the nomograms for predicting status of MVI. Clinical impact curves of the nomogram at each time point in the training cohort (**A**), internal validation cohort (**B**), and external validation cohort (**C**), respectively. At different threshold probabilities within a given population, the number of high-risk patients and the number of high-risk patients with the outcome were shown.
